# Factors associated with adequate antenatal care use among women of childbearing age in Burkina Faso: finding from the 2010 and 2021 demographic and health surveys

**DOI:** 10.3389/fpubh.2025.1526255

**Published:** 2025-04-16

**Authors:** Hermann Badolo, Aristide Romaric Bado, Herman Bazié, Yisso Fidèle Bacyé, Romaine Konseiga, Hervé Hien

**Affiliations:** ^1^Institut national de santé publique, Ouagadougou, Burkina Faso; ^2^IRSS, Centre national de la recherche scientifique et technologique, Ouagadougou, Burkina Faso; ^3^Centre Universitaire de Tenkodogo, Université Thomas Sankara, Ouagadougou, Burkina Faso; ^4^Centre Universitaire de Dori, Université Thomas Sankara, Ouagadougou, Burkina Faso

**Keywords:** antenatal care, health service use, associated factors, women of childbearing age, Burkina Faso

## Abstract

**Introduction:**

Antenatal care (ANC) is a critical determinant of maternal and infant wellbeing and is a reliable method for reducing maternal and infant mortality. Antenatal care use is considered adequate when the first ANC takes place in the first trimester and the woman completes at least four ANC in accordance with WHO recommendations during her pregnancy. Despite the increasing of the proportion of women having completed at least four ANC in Burkina Faso, the data show that WHO recommendations are far from being respected. This study aimed to determine the evolution of individual, family and community factors associated with the adequate use of ANC in Burkina Faso between 2010 and 2021.

**Methods:**

The data used in this study is procured from the Demographic and Health Surveys carried out in Burkina Faso in 2010 and 2021. Binary logistic regression analysis was used to analyze factors associated with the use of antenatal care. Adjusted odds ratios (AOR) were estimated to assess the strength of associations, and 95% confidence intervals were used for significance testing. A proportion test was used to examine differences in ANC utilization between 2010 and 2021 in Burkina Faso.

**Results:**

In our study sample, 22.92% (95% CI: 22.11–23.74) of the respondents in 2010 had adequate ANC use, compared to 46.34% (95% CI: 45.12–47.58) in 2021. The results demonstrate the influence of the woman’s individual characteristics, the household and the community characteristics on the adequate ANC use in Burkina Faso. Regarding the woman’s individual characteristics, age, educational level, marital status, occupation and modern contraceptive methods use were significantly associated with adequate ANC use in 2010 and 2021. The household wellbeing quintile, the degree of exposure to the media and the region of residence were significantly associated with adequate antenatal care use in 2010 and 2021.

**Conclusion:**

This study notes that Burkina Faso has made enormous progress in improving the coverage of antenatal care between 2010 and 2021, and indicates several factors including individual, family and community factors influencing adequate ANC use. For optimal efficacy, interventions promoting the adoption of antenatal care services must take these outcomes into account.

## Introduction

Maternal health, both globally in general and in developing countries in particular, is at the heart of national and international health sector policies. According to the World Health Organization (WHO), approximately 287,000 women died during or after pregnancy or childbirth in 2020 (1). Nearly 95% of these maternal deaths in 2020, most of which were preventable, occurred in low- and middle-income countries (1). In African countries, maternal mortality is one of the leading causes of death among women and newborns. The main complications and causes of pregnancy-related death are hemorrhage, sepsis, unsafe abortion, hypertension problems and dystocia (2, 3). Most of these deaths and complications could largely be avoided or treated during antenatal care, which aim to prevent, detect early and manage any complications that could affect the health of the mother and the unborn child, as well as support the woman throughout her pregnancy (2, 4). Available data indicate that countries with low ANC coverage tend to have high maternal mortality rates (5, 6). Additionally, it has been demonstrated that early use of antenatal care, starting in the first trimester of pregnancy, plays a critical role in the early detection and treatment of maternal health problems during pregnancy (7). This approach also constitutes a solid basis for adequate care during and after childbirth. Given the importance of ANC, the WHO recommends four antenatal l visits and at least eight contacts for pregnant women. Antenatal care use is considered adequate when the first ANC takes place in the first trimester and the woman completes at least four ANC in accordance with WHO recommendations during her pregnancy (4, 7).

In Burkina Faso, the maternal mortality ratio (MMR) is experiencing a downward trend, going from 341 deaths per 100,000 live births between 2003 and 2010 to 232 deaths per 100,000 live births between 2014 and 2021 (8–10). Despite this progress, difficulties persist regarding certain key indicators of maternal and child health. Indeed, according to reports from demographic and health surveys (DHS), since 2003, the proportion of women having completed at least four antenatal consultations during their pregnancy has increased from 17.6% in 2003 to 33.7% in 2010 and 72.1% in 2021. Furthermore, the proportion of women having undergone their first antenatal consultation during the first trimester of pregnancy also increased from 26.9% in 2003 to 41.2% in 2010 and 47.7% in 2021 (8–10). Despite this progress, these data show that WHO recommendations are far from being respected in Burkina Faso.

Studies conducted in Burkina Faso on antenatal healthcare services have predominantly concentrated on access to and utilization of antenatal care (ANC), investigating the factors that influence these dimensions, including socio-economic, geographical, and cultural determinants (11, 12). Additionally, they analyze the effects of free-of-charge policies on the utilization of maternal health services (13), as well as the repercussions of terrorist attacks and insecurity on access to maternal health services, encompassing ANC visits, assisted deliveries, and cesarean sections (14). Other researchers have assessed the quality and content of care received during ANC visits (15, 16). However, the adequate utilization of antenatal care among women of childbearing age in Burkina Faso has not been explicitly addressed, nor has a comparative analysis been conducted with the data available from the 2010 and 2021 Demographic and Health Surveys (DHS).

With a gross domestic product (GDP) of approximately 768.8 USD per capita in 2020 (17), Burkina Faso is one of the countries with the lowest level of economic development. More than 40% of its population live below the poverty line. The 2021–2022 report of the Human Development Index (HDI) of the United Nations Development Program (UNDP), ranks Burkina Faso 184th out of 191 countries. Burkina Faso’s economy is mainly based on agriculture and mining. Agriculture, which occupies nearly 82% of the active population, is currently hampered by the security context, which limits access to rural areas (17).

The health situation in Burkina Faso, although improving, is characterized by high general and specific mortality rates. Mothers and children constitute the most vulnerable groups. According to various DHSs carried out in Burkina Faso, the infant mortality rate decreased regularly between 1998 and 2021, going from 105 to 30%. Life expectancy at birth continues to increase. Overall, it increased from 42.2 years in 1975 to 61.9 years in 2019, an increase of 19.7 years (8–10, 18, 19).

In addition, in a difficult context marked by COVID-19 and security challenges, there was a general decline in the use of health services in 2020 compared to previous years in Burkina Faso. For example, at the national level, the coverage of the fourth ANC was 38.0% for a target of 60% in 2020. The extreme values were recorded in the Sahel (9.7%) and Cascades (49.3%) regions. None of the regions had reached the target in 2020 (20).

Building on existing research into antenatal care (ANC) utilization in Burkina Faso (11–16, 21–25), this study analyzes factors associated with adequate ANC use in 2010 and 2021 to inform strategies for reducing maternal and neonatal mortality.

The study’ objective is to identify sociocultural and economic determinants—including women’s living environment, reproductive behavior, and individual characteristics—that influence ANC uptake. We hypothesize that enhancing women’s social status (literacy, economic agency, decision-making autonomy), promoting positive reproductive behaviors (family planning, health service engagement), and improving maternal health practices could significantly increase adequate ANC utilization in Burkina Faso. In a country where public health services face a serious lack of equipment, medicines and competent personnel, this study could contribute to providing better guidance, information and awareness-raising activities for women in matters related to the use of antenatal care.

## Materials and methods

### Study setting

Burkina Faso, a landlocked nation situated in the Sahel region, covers an area of 272,969 km^2^ and is bordered by Mali to the northwest, Niger to the northeast, Benin to the southeast, and Côte d’Ivoire, Ghana, and Togo to the south. Administratively, the country is divided into 13 regions, 45 provinces, 351 departments, and 8,228 villages. According to the 2019 General Census of Population and Housing (RGPH), Burkina Faso’s population was recorded at 20.5 million, with women constituting 51.7% of this total (10.6 million). Notably, 44% of women (4.66 million) were of reproductive age (15–49 years), highlighting the importance of maternal healthcare services. Furthermore, the 0–4 age group comprised 16.2% of the population (3.3 million), emphasizing the demographic significance of neonatal health outcomes (26).

### Data sources

This study utilizes nationally representative data from the 2010 and 2021 Demographic and Health Surveys (DHS), conducted during periods characterized by ongoing maternal and neonatal health challenges. Burkina Faso’s decentralized administrative structure, along with the socioeconomic disparities across its regions, provides a critical context for analyzing spatial and temporal trends in antenatal care utilization. These data were collected from a representative sample of women of childbearing age (15–49 years), collected during these two household surveys. The DHS reports carried out in 2010 and 2021 provide more details on these surveys (8, 10).

### Study population and sample size

**Figure 1:**
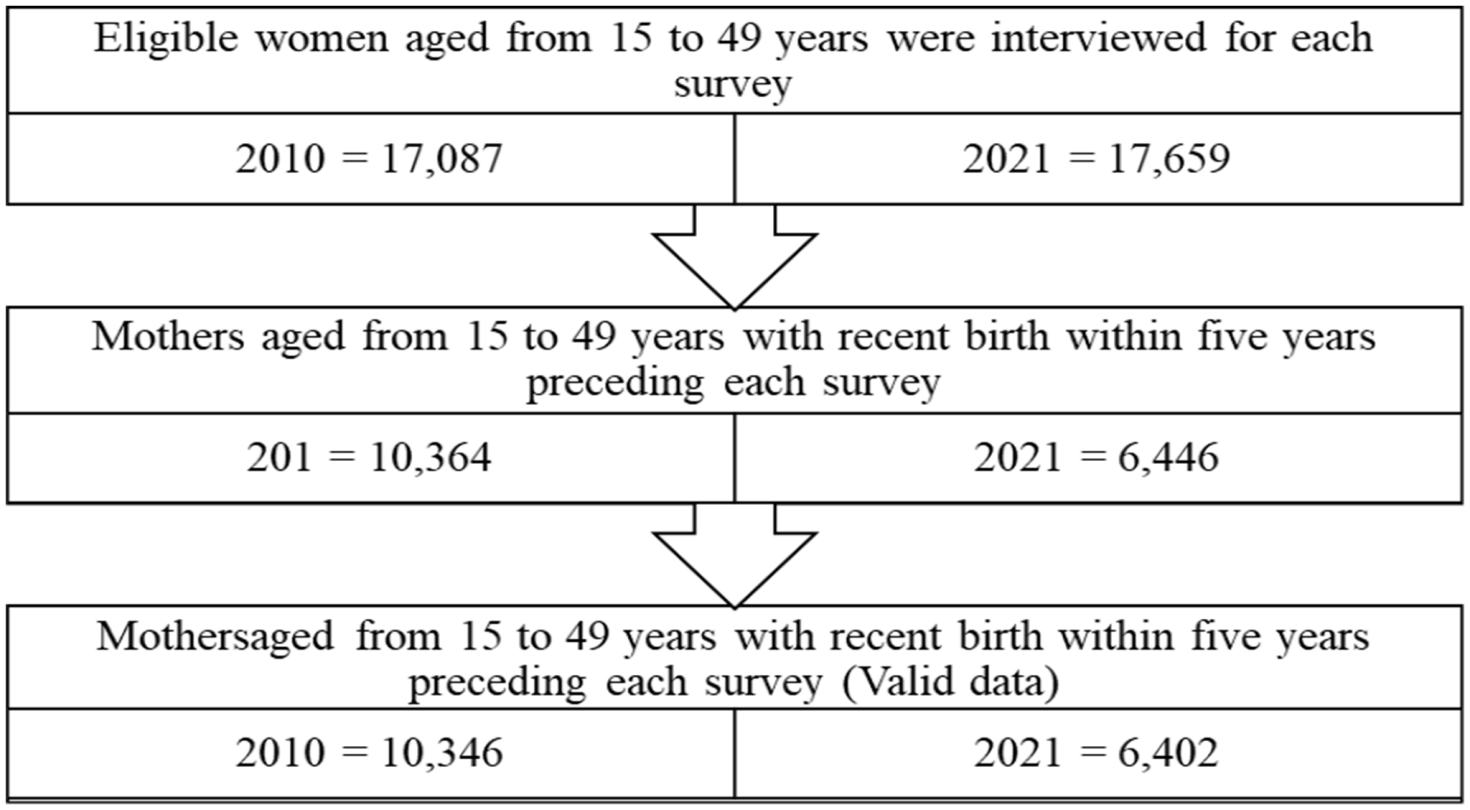
Flowchart showing how the final sample size was obtained.

The population studied consists of women of childbearing age, i.e., those aged from 15 to 49 years, who have had at least one live birth during the last 5 years preceding each of these two surveys. If a woman had more than one live birth within the past 5 years, only the utilization of ANC during the most recent birth was taken into account for data analysis in this study. The DHS sample only includes ordinary households, thus excluding populations in collective households such as hospitals, prisons, refugee camps and internally displaced people (Figure 1).

### Study variables

The variables under study were taken from the household questionnaire and the individual women’s questionnaire used during these two surveys.

### Dependent variable

The dependent variable is adequacy of antenatal care use, which is the measure of compliance with prescribed recommendations in terms of timing and frequency of antenatal use. This variable was constructed by combining:

- Timeliness: Timing of the first ANC visit (categorized as “adequate” if initiated in the first trimester);
- Frequency: Total ANC visits (categorized as “adequate” if ≥4 visits);

A dichotomous variable was created (1 = adequate if both criteria were met; 0 = otherwise). This variable was measured dichotomously and coded as 0 for inadequate and 1 for adequate.

### Independent variables

The independent variables used in the study were selected based on the results of previous research on the determinants of antenatal care utilization (27–32). At the individual level, this study considered the mothers’ age at the time of delivery, the use of modern contraceptive methods, the mothers’ education level, the mothers’ occupation, the mothers’ marital status, the mothers’ average parity and the interval between pregnancies. At the household level, this study considered the gender of the household head, the household wellbeing quintile, the size of the household and decision-making regarding the woman’s health. At the community level, variables on the degree of exposure to the media, the place of residence and the region of residence were considered.

### Statistical analysis

The data were cleaned, coded and analyzed using Stata version 16.1 software.

Regarding the objectives of this study, the analytical approach includes both a descriptive part and an explanatory part. In the descriptive part, a bivariate analysis made it possible to describe the relationships between the independent variables and the dependent variable. The chi-square (95%) test was used to identify possible statistical links between the dependent variable and the independent variables. The proportion test (95%) was used to examine differences in adequate ANC utilization between 2010 and 2021 in Burkina Faso.

To analyze factors associated with adequate ANC utilization, multivariate binary logistic regression analyses were used. AORs were estimated to assess the strength of associations and, 95% confidence intervals were used for significance testing. All analyses used sampling weights and adjusted for the sampling design (clustering and stratification) to take into account the complex sampling of DHS and to ensure representativeness.

## Results

### Sample description

This study included 10,346 women in 2010 and 6,402 women aged 15–49 years who had at least one live birth during the last 5 years preceding these two surveys (Table 1). In 2010, 24.0% (2,481/10,346) resided in urban areas, compared to 29.0% (1,855/6,402) in 2021. Adequate antenatal care (ANC) utilization increased significantly from 22.9% (95% CI: 22.11–23.74) in 2010 to 46.3% (95% CI: 45.12–47.58) in 2021 (*p* < 0.001).

**Table 1:** Descriptive results of the use of antenatal care among women aged 15–49 based on data from the 2010 and 2021 demographic and health surveys in Burkina Faso.

Characteristics	2010			2021			Proportion test of increase (*p*-value)
	n	% ANC adequate (95% CI)	*P*-value	n	% ANC adequate (95% CI)	*P*-value	
Total	10,346	22.92 (22.11–23.74)	–	6,402	46.34 (45.12–47.58)	–	*p* < 0.001
Mothers’ individual characteristics							
Mothers’ age							
15–19	592	18.75 (15.68–22.13)	0.002	529	38.56 (34.39–42.86)	0.0027	*p* < 0.001
20–24	2,404	24.96 (23.24–26.74)		1,613	44.64 (42.19–47.10)		*p* < 0.001
25–29	2,574	24.16 (22.52–25.87)		1,506	48.21 (45.66–50.77)		*p* < 0.001
30–34	2,165	22.73 (20.97–24-55)		1,340	48.66 (45.95–51.37)		*p* < 0.001
35–39	1,476	21.82 (19.73–24.01)		910	46.92 (43.64–50.23)		*p* < 0.001
40–44	873	20.05 (17.44–22.86)		414	47.34 (42.45–52.28)		*p* < 0.001
45–49	262	18.70 (14.16–23.93)		90	46.67 (36.07–57.49)		*p* < 0.001
Mothers’ educational level							
None	8,431	20.47 (19.62–21.35)	*p* < 0.001	4,260	44.53 (43.03–46.04)	*p* < 0.001	*p* < 0.001
Primary	1,243	29.04 (26.53–31.65)		933	45.02 (41.79–48.27)		*p* < 0.001
Secondary and more	672	42.26 (38.49–46.10)		1,209	53.76 (50.91–56.60)		*p* < 0.001
Mothers’ marital status							
Never married	168	17.86 (12.38–24.50)	0.057	191	31.41 (24.90–38.51)	0.0001	0.003
In union	9,949	22.89 (22.06–23.73)		6,108	46.84 (45.58–48.10)		*p* < 0.001
Divorced/Separated	229	27.95 (22.24–34.24)		103	44.66 (34.86–5,478)		0.003
Average parity of women							
1 child	1,783	27.76 (25.59–29.90)	*p* < 0.001	1,356	46.53 (43.85–49.23)	0.3934	*p* < 0.001
2 children	1,772	24.89 (22.89–26.97)		1,202	46.51 (43.66–49.37)		*p* < 0.001
3 children	1,576	24.24 (22.14–26.43)		1,116	47.58 (44.62–50.56)		*p* < 0.001
4 children and more	5,215	20.19 (19.11–21.31)		2,709	45.81 (43.92–47.71)		*p* < 0.001
Birth interval							
<2 years	823	18.47 (15.87–21.29)	*p* < 0.001	385	37.4 (32.55–42.45)	*p* < 0.001	*p* < 0.001
2–3 years	3,025	19.57 (18.17–21.03)		1,309	42.32 (39.63–45.05)		*p* < 0.001
3 years and above	6,498	25.04 (23.99–26.11)		4,708	48.19 (46.76–49.63)		*p* < 0.001
Mothers’ religion							
Without religion	118	15.25 (09.30–23.03)	*p* < 0.001	78	48.72 (37.23–60.31)	0.0010	*p* < 0.001
Muslim	6,394	21.94 (20.93–22.98)		4,156	45.74 (44.22–47.27)		*p* < 0.001
Christian	2,892	27.35 (25.73–29.02)		1,879	49.02 (46.73–51.30)		*p* < 0.001
Animist	942	16.88 (14.54–19.43)		289	37.02 (31.44–42.87)		*p* < 0.001
Mothers’ occupation							
Unemployed	1,802	20.98 (19.12–22.93)	*p* < 0.001	2,342	44.06 (42.04–46.10)	*p* < 0.001	*p* < 0.001
Qualified job	156	56.41 (48.25–64.32)		235	61.70 (55.16–67.95)		0.297
Trade	2,208	24.64 (22.85–26.49)		1,163	46.6 (43.71–49.52)		*p* < 0.001
Agriculture	5,333	21.70 (20.60–22.83)		2,317	46.53 (44.48–48.58)		*p* < 0.001
Manual job	774	23.00 (20.08–26.13)		319	48.59 (42.98–54.22)		*p* < 0.001
Use of contraceptive methods							
No method	8,605	21.13 (20.27–22.01)	*p* < 0.001	3,777	43.74 (42.15–45.34)	*p* < 0.001	*p* < 0.001
Methods traditional	110	37.27 (28.24–47.01)		178	34.27 (27.33–41.74)		0.605
Methods modern	1,631	31.39 (29.14–33.71)		2,447	51.25 (49.25–53.24)		*p* < 0.001
Household characteristics							
Gender of household head							
Male	9,632	22.81 (21.97–23.66)	0.339	5,848	45.96 (44.68–47.25)	0.0473	*p* < 0.001
Female	714	24.37 (21.26–27.69)		554	50.36 (46.11–54.60)		*p* < 0.001
Household size							
1–3 persons	1,196	25.33 (22.89–27.90)	0.045	515	50.29 (45.88–54.69)	0.0086	*p* < 0.001
4–5 persons	2,533	23.92 (22.27–25.63)		1,340	48.28 (45.58–50.99)		*p* < 0.001
6–9 persons	3,933	21.97 (20.68–23.30)		2,474	46.77 (44.78–48.76)		*p* < 0.001
10 persons and above	2,684	22.28 (20.72–23.90)		2,073	43.61 (41.46–45.77)		*p* < 0.001
Household wellbeing quintile							
Very poor	1,896	16.35 (14.71–18.09)	*p* < 0.001	1,249	43.23 (40.47–46.04)	*p* < 0.001	*p* < 0.001
Poor	2,096	17.99 (16.36–19.70)		1,279	45.11 (42.36–47.89)		*p* < 0.001
Average	2,225	22.29 (20.58–24.08)		1,399	45.03 (42.40–47.68)		*p* < 0.001
Rich	2,264	25.27 (23.49–27.11)		1,365	44.98 (42.32–47.67)		*p* < 0.001
Very rich	1,865	33.03 (30.90–35.22)		1,110	54.59 (51.61–57.55)		*p* < 0.001
Decision-making regarding the health of women in the household							
Mother alone	712	26.54 (23.33–29.95)	0.008	655	42.90 (39.07–46.79)	*p* < 0.001	*p* < 0.001
Mother and her partner	1,553	24.98 (22.85–27.22)		1,121	52.63 (49.66–55.59)		*p* < 0.001
Partner alone	7,605	22.12 (21.19–23.07)		4,249	45.87 (44.36–47.38)		*p* < 0.001
Others	79	22.78 (14.10–33.60)		83	49.40 (38.24–60.60)		*p* < 0.001
Characteristics of the living environment							
Place of residence							
Urban	2,481	29.75 (27.95–31.59)	*p* < 0.001	1855	48.36 (46.06–50.66)	0.0393	*p* < 0.001
Rural	7,865	20.76 (19.87–21.68)		4,547	45.52 (44.07–46.99)		*p* < 0.001
Region of residence							
Boucle du Mouhoun	893	16.46 (14.09–19.06)	*p* < 0.001	519	43.55 (39.23–47.93)	*p* < 0.001	*p* < 0.001
Cascades	665	19.10 (16.18–22.30)		311	54.02 (48.30–5,966)		*p* < 0.001
Centre	696	35.34 (31.79–39.02)		598	43.31 (39.30–47.39)		0.003
Central-Est	807	38.79 (35.41–42.25)		618	52.43 (48.41–56.43)		*p* < 0.001
Central-Nord	779	19.00 (16.30–21.93)		466	59.66 (55.05–64.14)		*p* < 0.001
Central-Ouest	909	20.35 (17.78–23.12)		541	38.26 (34.15–42.51)		*p* < 0.001
Central-Sud	707	30.98 (27.58–34.52)		392	41.07 (36.16–46.12)		0.001
Est	979	25.03 (22.34–27.86)		418	55.50 (50.59–60.33)		*p* < 0.001
Hauts-Bassins	870	22.76 (20.01–25.69)		651	48.08 (44.18–51.99)		*p* < 0.001
Nord	818	19.93 (17.24–22.83)		518	54.25 (49.86–58.60)		*p* < 0.001
Plateau Centrale	723	18.67 (15.90–21.71)		577	46.45 (42.32–50.61)		*p* < 0.001
Sahel	784	09.82 (07.83–12.12)		263	19.01 (14.45–24.28)		*p* < 0.001
Sud-Ouest	716	23.46 (20.40–26.74)		530	37.74 (33.59–42.02)		*p* < 0.001
Degree of media exposure							
Null	2,667	19.05 (17.57–20.59)	*p* < 0.001	2,079	44.20 (42.05–46.37)	*p* < 0.001	*p* < 0.001
Weak	5,772	21.66 (20.60–22.74)		2,674	45.44 (43.54–47.35)		*p* < 0.001
Average	1,604	28.99 (26.78–31.28)		1,529	50.36 (47.82–52.90)		*p* < 0.001
High	303	48.84 (43.09–54.63)		120	52.50 (43.18–61.69)		0.498

### Bivariate descriptive analysis of the factors of use of antenatal services

Table 1 presents the percentages of women aged 15–49 who carried out adequate ANC use during their last pregnancy in 2010 and 2021. We note that, whatever the year, the individual characteristics of the woman (age, educational level, marital status, occupation, religion, average parity and pregnancy interval), household characteristics (size, Household wellbeing quintile and decision-making regarding the health of the woman) and community characteristics (place of residence, region of residence and degree of exposure to the media) were significantly associated with adequate ANC use. However, gender of household head in 2010 and average parity in 2021 were not significantly associated with adequate ANC use.

Between 2010 and 2021, the utilization of antenatal care (ANC) during pregnancy improved markedly across most demographic groups within the study population, indicating progress in maternal health equity. While women in skilled occupations, traditional contraceptive users, and those with high media exposure exhibited no significant changes, historically marginalized groups experienced notable gains. Among mothers aged 45–49 years, the completion rate of adequate ANC rose from 18.70% (95% CI: 14.16–23.93) to 46.67% (95% CI: 36.07–57.49)—a nearly 2.5-fold increase. Similarly, multiparous women (those with four or more children) witnessed their ANC coverage more than double, increasing from 20.19% (95% CI: 19.11–21.31) to 45.81% (95% CI: 43.92–47.71).

Socioeconomic disparities also narrowed during this period. Mothers engaged in manual labor, for instance, increased their ANC utilization from 23.00% (95% CI: 20.08–26.13) to 48.59% (95% CI: 42.98–54.22), while those in female-headed households progressed from 24.37% (95% CI: 21.26–27.69) to 50.36% (95% CI: 46.11–54.60). Notably, mothers in very poor households—a demographic often disproportionately excluded from healthcare—achieved a rise from 16.35% (95% CI: 14.71–18.09) to 43.23% (95% CI: 40.47–46.04). Even non-religious mothers, a demographic frequently overlooked in health interventions, saw their ANC completion rates surge from 15.25% (95% CI: 9.30–23.03) to 48.72% (95% CI: 37.23–60.31).

Paradoxically, access to media did not uniformly predict improvements: mothers without media exposure still doubled their ANC utilization (19.05% [95% CI: 17.57–20.59] to 44.20% [95% CI: 42.05–46.37]). These trends collectively underscore a shift toward broader accessibility of ANC over the decade. However, persistent gaps among skilled workers, traditional method users, and women exposed to media suggest that systemic barriers—such as workplace constraints, cultural preferences, or fragmented health messaging—may require targeted policy attention.

### Factors associated with adequate antenatal care utilization

The results of the multivariate logistic regression are presented in Table 2. Maternal age, education, and contraceptive use were consistently associated with adequate antenatal care (ANC) utilization across both survey years. Women aged 20–44 years exhibited significantly higher odds of adequate ANC use compared to adolescents (15–19 years), with the strongest association in 2021 (AOR = 1.68, 95% CI:1.18–2.40 for ages 40–44). Secondary education or higher increased the likelihood of adequate ANC utilization in both 2010 (AOR = 1.48, 95% CI:1.16–1.88) and 2021 (AOR = 1.38, 95% CI:1.16–1.64). Modern contraceptive use remained a robust predictor (2010: AOR = 1.29, 95% CI:1.13–1.48; 2021: AOR = 1.34, 95% CI:1.20–1.50).

**Table 2:** Factors associated with the use of antenatal care among women aged 15–49 based on data from the 2010 and 2021 demographic and health surveys in Burkina Faso.

Caractéristiques	2010			2021		
	n	AOR (95% IC)	*P*-value	n	AOR (95% IC)	*P*-value
Mothers’ individual characteristics						
Mothers’ age						
15–19	592	1		529	1	
20–24	2,404	1.385 (1.072–1.791)	0.013	1,613	1,256 (0.99–1.593)	0.052
25–29	2,574	1.393 (1.048–1.852)	0.022	1,506	1.549 (1.182–2.031)	0.001
30–34	2,165	1.317 (0.966–1.797)	0.082	1,340	1.675 (1.245–2.254)	0.001
35–39	1,476	1.315 (0.946–1.83)	0.103	910	1.600 (1.162–2.201)	0.003
40–44	873	1.233 (0.867–1.755)	0.244	414	1.681 (1.177–2.399)	0.004
45–49	262	1.135 (0.725–1.778)	0.579	90	1.630 (0.958–2.773)	0.049
Mothers’ educational level						
None	8,431	1		4,260	1	
Primary	1,243	1.206 (1.034–1.407)	0.017	933	1.036 (0.884–1.214)	0.775
Secondary and above	672	1.476 (1.161–1.878)	0.002	1,209	1.382 (1.164–1.640)	*p* < 0.001
Mothers’ marital status						
Never married	168	1		191	1	
In union	9,949	1.365 (0.917–2.032)	0.803	6,108	1.929 (1.415–2.629)	0.002
Divorced/Separated	229	1.784 (1.094–2.910)	0.034	103	1.762 (1.075–2.888)	0.016
Mothers’ average parity						
1 child	1783	0.842 (0.693–1.022)		1,356	1	
2 children	1772	0.827 (0.664–1.03)	0.082	1,202	0.951 (0.776–1.164)	0.097
3 children	1,576	0.772 (0.609–0.979)	0.09	1,116	0.882 (0.700–1.110)	0.121
4 children & above	5,215		0.033	2,709	0.853 (0.663–1.096)	0.16
Birth interval						
<2 years	823	1		385	1	
2–3 years	3,025	1.026 (0.835–1.261)	0.806	1,309	1.203 (0.942–1.536)	0.142
3 years and above	6,498	1.200 (0.982–1.465)	0.074	4,708	1.453 (1.16–1.821)	0.002
Mothers’ religion						
Without religion	118	1		78	1	
Muslim	6,394	1.493 (0.881–2.53)	0.137	4,156	0.673 (0.413–1.097)	0.115
Christian	2,892	1.624 (0.958–2.752)	0.072	1879	0.780 (0.476–1.276)	0.32
Animist	942	1.069 (0.613–1.863)	0.814	289	0.622 (0.363–1.066)	0.082
Mothers’ occupation						
Unemployed	1802	1		2,342	1	
Qualified job	156	2.066 (1.376–3.103)	*p* < 0.001	235	1.484 (1.079–2.042)	0.01
Trade	2,208	0.991 (0.838–1.172)	0.916	1,163	1.011 (0.861–1.188)	0.817
Agriculture	5,333	1.172 (1.00–1.373)	0.049	2,317	1.045 (0.915–1.193)	0.496
Manual job	774	1.181 (0.93–1.499)	0.172	319	1.137 (0.878–1.471)	0.333
Use of contraceptive methods						
No method	8,605	1		3,777	1	
Traditional methods	110	1.424 (0.932–2.177)	0.102	178	0.667 (0.479–0.930)	0.016
Modern methods	1,631	1.294 (1.132–1.479)	*p* < 0.001	2,447	1.339 (1.197–1.498)	*p* < 0.001
Household characteristics						
Gender of household head						
Male	9,632	1		5,848	1	
Female	714	0.962 (0.774–1.195)	0.738	554	1.260 (1.026–1.548)	0.02
Household size						
1–3 persons	1,196	1		515	1	
4–5 persons	2,533	1.032 (0.85–1.251)	0.76	1,340	0.94 (0.743–1.190)	0.653
6–9 persons	3,933	1.050 (0.867–1.271)	0.621	2,474	0.921 (0.734–1.154)	0.564
10 persons & above	2,684	1.103 (0.901–1.35)	0.345	2073	0.889 (0.705–1.122)	0.362
Household wellbeing quintile						
Very poor	1896	1		1,249	1	
Poor	2096	1.104 (0.928–1.314)	0.269	1,279	0.979 (0.827–1.160)	0.817
Average	2,225	1.300 (1.096–1.542)	0.003	1,399	1.028 (0.865–1.221)	0.745
Rich	2,264	1.483 (1.246–1.766)	*p* < 0.001	1,365	1.039 (0.857–1.259)	0.636
Very rich	1865	1.560 (1.24–1.962)	*p* < 0.001	1,110	1.603 (1.236–2.079)	*p* < 0.001
Decision-making regarding the mother health in the household						
Mother alone	712	1		655	1	
Mother and her partner	1,553	1.046 (0.842–1.300)	0.685	1,121	1.490 (1.214–1.830)	*p* < 0.001
Partner alone	7,605	1.011 (0.835–1.224)	0.913	4,249	1.173 (0.983–1.40)	0.063
Others	79	1.108 (0.623–1.969)	0.732	83	1.169 (0.721–1.896)	0.414
Characteristics of the living environment						
Place of residence						
Urban	2,481	1		1855	1	
Rural	7,865	1.093 (0.938–1.275)	0.259	4,547	1.226 (1.041–1.445)	0.013
Region of residence						
Boucle de Mouhoun	893	1		519	1	
Cascades	665	1.073 (0.814–1.414)	0.634	311	1.351 (0.99–1.844)	0.064
Centre	696	1.845 (1.422–2.393)	*p* < 0.001	598	0.674 (0.512–0.887)	0.005
Centre-Est	807	3.315 (2.607–4.215)	*p* < 0.001	618	1.423 (1.105–1.833)	0.006
Centre-Nord	779	1.144 (0.88–1.488)	0.309	466	2.112 (1.615–2.762)	*p* < 0.001
Centre-Ouest	909	1.247 (0.969–1.604)	0.086	541	0.758 (0.583–0.987)	0.038
Centre-Sud	707	2.085 (1.62–2.683)	*p* < 0.001	392	0.786 (0.592–1.045)	0.1
Est	979	1.951 (1.526–2.495)	*p* < 0.001	418	1.677 (1.272–2.213)	*p* < 0.001
Hauts Basins	870	1.23 (0.954–1.586)	0.113	651	1.128 (0.875–1.455)	0.391
Nord	818	1.24 (0.958–1.605)	0.103	518	1.517 (1.169–1.97)	0.002
Plateau Central	723	1.032 (0.789–1.35)	0.818	577	1.118 (0.865–1.444)	0.405
Sahel	784	0.681 (0.495–0.938)	0.019	263	0.302 (0.207–0.441)	*p* < 0.001
Sud-Ouest	716	1.915 (1.435–2.555)	*p* < 0.001	530	0.800 (0.599–1.067)	0.141
Degree of media exposure						
Null	2,667	1		2079	1	
Weak	5,772	1.226 (1.083–1.387)	0.001	2,674	0.986 (0.869–1.12)	0.844
Average	1,604	1.406 (1.173–1.685)	*p* < 0.001	1,529	1.080 (0.92–1.269)	0.376
High	303	1.767 (1.263–2.474)	0.001	120	0.782 (0.512–1.195)	0.26

Household wealth quintile significantly influenced ANC utilization, though its impact narrowed by 2021. In 2010, even average wealth (AOR = 1.30, 95% CI:1.10–1.54) increased ANC use, but by 2021, only the *very rich* quintile showed a strong association (AOR = 1.60, 95% CI:1.24–2.08). Female-headed households in 2021 had higher ANC utilization (AOR = 1.26, 95% CI:1.03–1.55). In 2010, individual autonomy played a central role in ANC utilization. However, by 2021, shared decision-making between partners became a more significant predictor (AOR = 1.49, 95% CI: 1.21–1.83). Geographic inequities persisted, with women in the Sahel region facing significantly lower odds of adequate ANC utilization in both 2010 (AOR = 0.68, 95% CI:0.50–0.94) and 2021 (AOR = 0.30, 95% CI:0.21–0.44) compared to Boucle du Mouhoun. Conversely, the Centre-Est region showed consistently higher utilization (2010: AOR = 3.32, 95% CI:2.61–4.22; 2021: AOR = 1.42, 95% CI:1.11–1.83), likely due to concentrated health infrastructure. Rural residence became a positive predictor in 2021 (AOR = 1.23, 95% CI:1.04–1.45), suggesting potential improvements in rural healthcare access.

Meanwhile, the role of media exposure as a driver of ANC utilization diminished. In 2010, women with high media exposure were significantly more inclined to seek ANC services (AOR = 1.77, 95% CI: 1.26–2.47). By 2021, however, this association had dissipated.

## Discussion

The results indicate a significant improvement in access to adequate ANC consultations for pregnant women in Burkina Faso between 2010 and 2021. The results demonstrate the influence of mothers’ individual characteristics, household and community characteristics on the adequate ANC use in Burkina Faso. Concerning the mothers’ individual characteristics, her age, educational level, marital status, occupation and use of modern contraceptive methods were significantly associated with adequate ANC use in 2010 and 2021. The results of this study show a significant association between household characteristics (household wellbeing quintile, degree of media exposure and region of residence) and adequate ANC utilization in 2010 and 2021. The gender of the household head, decision-making regarding the mother’s health and the place of residence were significantly associated with adequate ANC use in 2021 only.

It is well recognized that women’s age plays an important role in the utilization of maternal health services (33, 34). In this study, middle-aged mothers were more likely to have adequate ANC use visits than younger and older mothers.

It appears in this study and in others (27, 32, 34–38) that women who have a low education level, encounter more difficulties in ensuring adequate use of antenatal care. This can be explained by the fact that less educated women often encounter significant challenges in acquiring knowledge about their health, comprehending the medical system, and understanding the mechanisms of antenatal care. This lack of awareness constitutes a critical factor contributing to delays in accessing the first antenatal care appointment, as many of these women remain uninformed regarding the appropriate timing and objectives of antenatal care (39).

Mothers’ occupation appears to have a very strong influence on their use of ANC services. The results of this study showed that working mothers were more likely to make multiple antenatal visits than unemployed mothers. This conclusion is consistent with that of Assefa and Tadesse (40), Tawiah (41), Sharma et al. (42), as well as Badolo (22). These results could be explained by the fact that working pregnant women have a larger social network which could allow them to access more information relating to pregnancy, including antenatal care. The information provided by their coworkers and that obtained in their workplace increased their knowledge about pregnancy, which ultimately enabled them to perform antenatal visits as recommended (43).

Household wellbeing quintile was strongly and positively associated with the use of ANC services. The findings indicated that women living in impoverished households were less likely to engage in adequate ANC utilization compared to their counterparts in rich households. Similar results were reported in previous studies conducted in Uganda (44), Ethiopia (27), and Nepal (33). These studies indicated that women living in very poor or poor households sought ANC services less frequently than those living in wealthier households.

Our findings indicate a significant positive association between media exposure and the utilization of antenatal care (ANC) in 2010; however, this association was no longer significant by 2021. This change may be attributed to improvements in access to health services in Burkina Faso between 2010 and 2021, which likely reduced reliance on media to raise awareness among women regarding pregnancy monitoring. Notably, since 2016, the government has undertaken a substantial recruitment initiative for trained community-based health workers with a commendable level of education, aimed at ensuring that populations have access to quality health services (45). These community-based health workers are now equipped to treat common childhood illnesses, provide family planning advice, and assist pregnant women in accessing appropriate healthcare. By 2024, the coverage of administrative villages by pairs of community-based health workers reached 93.08%. This community-based initiative has undoubtedly contributed to enhancing awareness of health issues among both urban and rural populations, as well as to an increased utilization of maternal health care services.

While our analysis highlights mothers’ individual factors, household and community factors associated with adequate ANC, implementation of the policy of free care for mothers and children such as the Gratuité programme (46) and other health financing reforms (e.g., performance-based financing) may have contributed to observed trends. These substantials health policy marked a pivotal moment in maternal health by eliminating financial barriers that previously hindered access to care. These policies of providing free healthcare may contribute significantly to the attainment of the Sustainable Development Goals (SDGs) concerning maternal and child health. Since its introduction, data reveal a notable transformation. Women, who were formerly constrained by financial considerations or geographical distance, are now seeking healthcare services earlier and more frequently. In their study, Offosse et al. demonstrated that 72% of women now attend at least four antenatal check-ups, facilitated by the provision of free healthcare, while 53% initiate these check-ups as early as the first trimester of pregnancy, representing a remarkable increase compared to previous years (13). However, these policies were not directly measured in our models. Future studies should incorporate these policies implementation data to disentangle their effects.

This study has some limitations. The data used is procured from the DHS, which use a robust multistage probability sampling methodology to select clusters and households. The first limitation lies in the cross-sectional nature of the data collected, which means that the dependent and explanatory variables were measured simultaneously and therefore cannot guarantee any causality of the associations. Additionally, the 5-year recall period may seem long and could question the validity of the responses but limiting it to the most recent births would reduce this risk. However, in African contexts, some births occur before the age of 15 years. The operational definition of reproductive age limited by default to the interval of 15 to 49 years, limits the actual population subject to maternity events. Some cultural variables, potentially relevant to explaining the adequacy of antenatal care, were not considered in this study because they were missing from the database. Also, the smaller subgroups (e.g., mothers aged 45–49, women with secondary education) may have reduced statistical power and the findings for these groups should be interpreted with caution.

## Conclusion

In sum, we note that Burkina Faso has made enormous progress in improving the coverage of antenatal care between 2010 and 2021. This has highlighted factors that influence the adequate ANC use, both at the individual, household and community level. At the individual level, age, occupation, education level and use of modern contraceptive methods were factors significantly associated with adequate ANC use. At the household and community level, living in a female-headed household, living in a wealthy household, living in a rural area, decision-making in the household in relation to the mother’s health and region of residence, were significantly associated with adequate ANC monitoring. To achieve optimal efficacy, interventions aimed at promoting the adequate ANC use must take these finding into account.

The findings of this study have important implications for the design of health policy regarding maternal health in Burkina Faso. Policies that encourage women’s use of modern contraceptive methods and promote women’s participation in the labor market could likely help increase attendance at antenatal care services. Therefore, government policies should target uneducated, unemployed and economically disadvantaged women to increase the rate of adequate ANC use.

## Data Availability

The datasets presented in this study can be found in online repositories. The names of the repository/repositories and accession number(s) can be found in the article/supplementary material.
